# Geoengineering: proactive measures to tackle climate change

**DOI:** 10.1093/nsr/nwad271

**Published:** 2023-10-25

**Authors:** He Zhu

**Affiliations:** Editorial Office of NSR, Beijing, China

## Abstract

As of 20 August, 2023, extensive parts of northern China including the capital city of Beijing had experienced arguably the hottest summer in recent years, recording consecutive days of high temperatures at or near 40°C. Against this backdrop, *National Science Review* (NSR) organized a web forum on a climate-related topic that needs more exposure to Chinese public and researchers alike: geoengineering. Compared to commonly-known methods to address climate change such as emission reduction, geoengineering proposes to take much more proactive measures such as injecting aerosol into the stratosphere to increase solar reflection, implementing iron fertilization in the ocean to promote microbial growth, and capturing and liquifying CO_2_ and injecting it directly into exhausted oil fields. The forum was hosted by Prof. Tong Zhu of Peking University with four panelists. Together, they introduced the scientific backgrounds and implementations of these proactive measures and discussed the pros and cons anticipated by researchers so far.

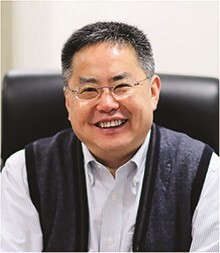

Fei Chai (柴扉)

Professor, College of Ocean and Earth Sciences, Xiamen University

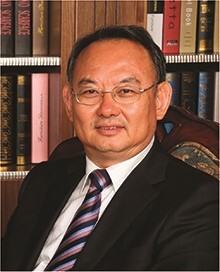

Zhijun Jin (金之钧)

Professor, SONOPEC Petroleum Exploration and Production Research Institute; Dean, Institute of Energy, Peking University; Dean, Peking University Ordos Research Institute of Energy

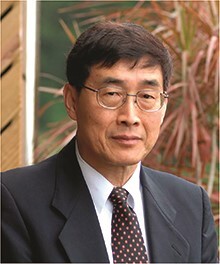

Shawchen Liu (刘绍臣)

Professor, Institute for Environmental and Climate Research, Jinan University

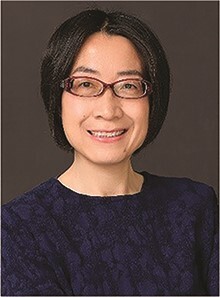

Jianhua Xu (徐建华)

Associate Professor, Department of Environmental Management, College of Environmental Sciences and Engineering and Institute for Global Health and Development, Peking University

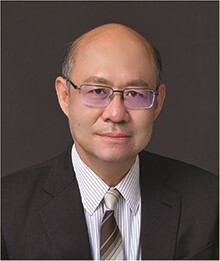

Tong Zhu (朱彤) (Chair)

Professor, College of Environmental Sciences and Engineering, Peking University

**Zhu**: First of all, I would like to thank today's panelists that joined in from all across China and abroad to participate in this forum. As we all know, geoengineering as a research topic has been gaining attention globally. Although it has been discussed for many years, it is still a topic of controversy. Possible implementations of geoengineering may create complicated economical, ecological and ethical issues. In order to explore these issues in depth, we invited leading experts who either have conducted relevant research or have participated in actual trials of geoengineering. We have Prof. and Academician Zhijun Jin of Peking University who specializes on carbon capture and sequestration (CCS); Prof. Shawchen Liu of Jinan University who focuses on injection of aerosol into the stratosphere; Prof. Fei Chai of Xiamen University who specializes in iron fertilization in the ocean; Prof. Jianhua Xu of Peking University who has been involved in research on the social aspects of geoengineering. To start, Prof. Liu, would you like to introduce the stratospheric aerosol injection for the panel?

I think that China has a considerable advantage in pushing for grand scale engineering projects such as this (aerosol injection) and it will improve our global standing in the efforts to address climate change.

— Shawchen Liu

**Liu**: According to reports from the US and the EU, one of the most effective and economical methods of geoengineering discovered so far is aerosol injection into the stratosphere to increase solar reflection and reduce heating at the surface. As Prof. Zhu pointed out, this method will create benefits as well as negative effects, like any other engineering endeavors. The key aspect is the balance between benefits and negative effects. Specifically, the aerosol injection method will have the most immediate effect of all geoengineering methods: it can take effect in a year or two. Another important advantage is that there have been previous volcanic aerosol injections into the stratosphere (such as the Pinatubo eruption in 1991 shown in Figure 1) for scientists to evaluate quantitatively their effectiveness, benefits and negative effects. It will certainly have risks, but fortunately the injected aerosols will dissipate in a year or two naturally from the stratosphere. So in the case of serious negative effects, the injection can be terminated in a timely manner.

**Figure 1.**
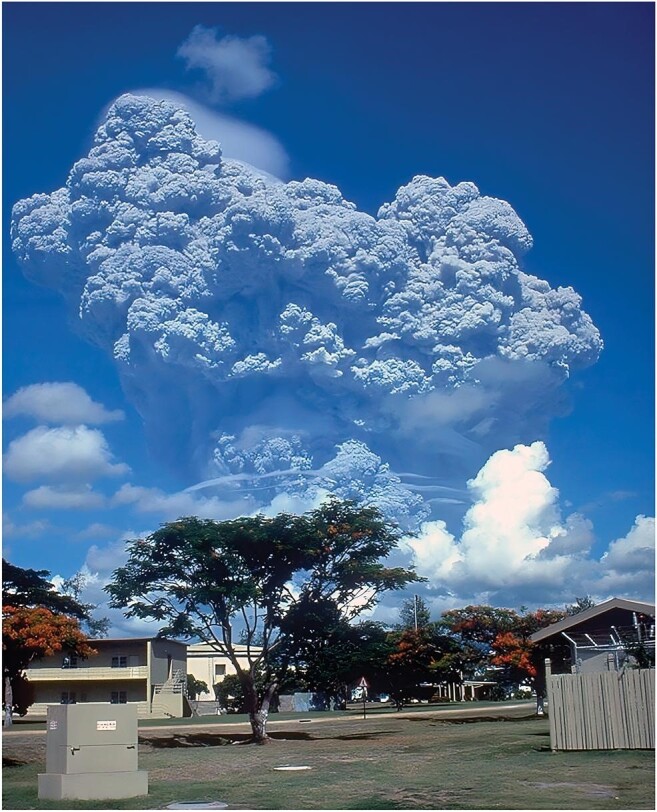
Eruption of Volcano Pinatubo in the Philippines in 1991 (credit: Wikipedia).

Volcano Pinatubo in the Philippines erupted in 1991. In the following two years the global temperature was reduced by about 0.5°C, which was about half of the amount of global warming at this moment. Possible negative effects in terms of acid rain and damage to stratospheric ozone were negligible according to the measurements made in China and elsewhere. So we may say that we have quantitative knowledge of the expected effects and risks of aerosol injections from past volcanic eruptions. Specifically, current estimates show that an injection of 20 megatons of sulfates into the stratosphere can reduce global temperature by approximately 1°C. Economically, 20 megatons is feasible. If we move forward, we will see effects in a year or two. If something goes wrong, we can stop the injection and the aerosols will dissipate naturally in about two years. However, this method is currently facing stiff resistance internationally. The main objection originates from its negative effects on emission reduction efforts of greenhouse gas that are already underway. Nevertheless, in my opinion, this is an option we cannot afford to ignore when we are facing the risk of global temperature rising substantially above 2°C by the end of the century with the current Intended Nationally Determined Contributions (INDCs) to reduce global greenhouse gas emission. A realistic estimate at this moment is that we are on track to get an approximate rise of 4°C by 2100. Therefore, we need an effective geoengineering project to slow down global warming and buy valuable time for global efforts in greenhouse gas emission reduction. We propose submitting to the United Nations a feasibility study on the injection of aerosols into the stratosphere. If the UN can agree on the balance between benefits and risks, we can start with a relatively small 2 megaton injection, aiming for a 0.1°C reduction as a proof of feasibility. I think that China has a considerable advantage in pushing for grand scale engineering projects such as this and it will improve our global standing in the efforts to address climate change.

**Zhu**: Thank you, Prof. Liu. Next topic is iron fertilization. Prof. Chai is an expert on ocean-based geoengineering. Prof. Chai, would you like to introduce it?

Moving forward, we are looking at multiple ways of implementing OIF experiments, laying out various extents of utility, efficiency, durability and impacts of near term and long term on the global scale.

— Fei Chai

**Chai**: Generally speaking, geoengineering can be divided into two categories: solar radiation management (SRM) and carbon dioxide removal (CDR). CDR consists of terrestrial based and ocean based methods. Terrestrial based methods are relatively mature, in terms of large-scale carbon sinks and re-forestation. Most of ocean-based CDR methods only started to gain attention in the past few years. One of the early methods is ocean iron fertilization (OIF) in the high nutrients and low chlorophyll (HNLC) regions. The first open ocean experiment was conducted in the eastern equatorial Pacific Ocean in 1992. During the past 30 years, there were a total of 14 OIF experiments in HNLC regions, in which a lot of information and new knowledge were accumulated. All the experiments suggested that OIF is an effective way to elevate phytoplankton growth and increase primary productivity. There are still some questions and issues related to the efficiency of carbon removal and the negative impact on marine ecosystems using OIF. For example, how much of carbon was transported to 500 meters or 1000 meters below? We do not have a clear answer for this question. The reason is simple: we did not have adequate technology and tools to track and measure carbon export to deep ocean in the previous studies. Some environmental groups also emphasized the negative side-effects of OIF, and they wanted to stop OIF experiments completely.

Now, we need to focus on the recent development of observational technologies to track and measure carbon export throughout the water column, from the surface to 1000 meters below surface and deeper. An ongoing effort of Measurement, Reporting and Verification (MRV) will be the goal for all ocean-based CDR methods through integrating sensor technologies, multiple observational platforms, and numerical modeling. The timing is right to combine ocean and environmental sciences with modern technology. However, to succeed, we need to work with legal and ethics communities specializing in international affairs, gaining the trust of the general public.

The other methods are relatively new in comparison to OIF. For example, alkalinity enhancement is a chemistry-based method to alter the ocean carbonate system in order to reduce partial pressure of CO_2_ at the ocean surface, however it may not directly affect ecological balance as much as iron fertilization. Some other methods include large-scale macroalgae farming. In this area, China's contribution to world total of macroalgae production is about 60%, and we mostly grow it as a food source. When we grow macroalgae as potential CDR, it will have to be in deep waters of open ocean, and it needs to sink to the bottom of the ocean as a method of permanent carbon storage.

In comparison with all ocean-based CDR methods, iron fertilization is the most mature. In the past two years, international efforts have been resumed to reorganize the ideas and past experiences in this area (Figure 2) and formed a consortium called Exploring Ocean Iron Solutions (ExOIS: https://oceaniron.org/). In this organization, we discuss the latest developments in science and technology as well as legal, social, and ethical issues. For example, who should be in charge these OIF experiments in the ocean? What are the codes of conduct and best practice for conducting OIF experiments? Moving forward, we are looking at multiple ways of implementing OIF experiments, laying out various extents of utility, efficiency, durability and impacts of near term and long term on the global scale. Chinese scientists in this field have accumulated extensive knowledge and we are in a strong position to join global efforts to investigate OIF as an effective ocean-based CDR method.

**Figure 2.**
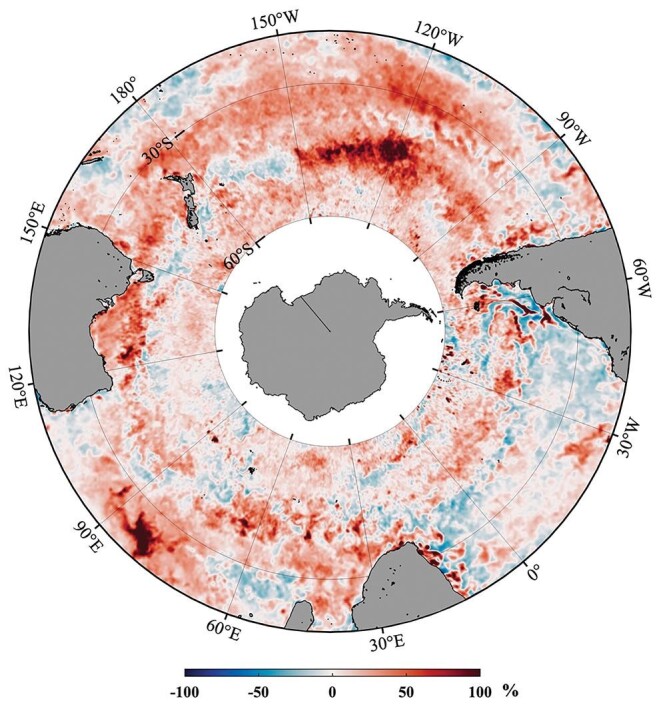
Map of averaged changing rate of sea surface chlorophyll induced by the 2019–2020 Australian bushfires (credit: Science of the Total Environment, 807, 150775. Used by permission).

**Zhu**: Thank you. Now, Prof. Jin, would you like to make some comments from your perspective?

So, from a global point of view, economic development should remain the theme and carbon emission is unavoidable. We should invest in CCS and SRM to reduce CO_2_ and temperature.

— Zhijun Jin

**Jin**: First of all, I think the forum we are having today is of great value and significance. Second, I want to concur with what Prof. Liu said in the beginning: based on the latest projection, it’d be difficult to limit temperature rise below 2°C by the end of this century. I think it is a wise choice to consider some artificial interventions of climate change. Although there is an enormous amount of research to be conducted, we have to start somewhere. So when Prof. Zhu invited me to this forum, I was quite eager to participate. I have learned a lot from what the experts before me had said. But allow me to start by answering a question: after algae growth, is it possible to preserve it as a permanent storage of carbon? Based on my professional knowledge in oil and gas exploration the answer is undoubtedly, yes. A primary source of oil and natural gas we extract today is alga from ancient oceans. So, if humans stop retrieving oil and gas as energy sources in the future, the alga grown and stored today will permanently remove CO_2_ from the atmosphere. I am personally optimistic about iron fertilization, given that we pay proper attention to its possible environmental and ecological impacts. My hometown, Tsingtao, a few years ago experienced an environmental crisis caused by *ulva prolifera*, commonly known as green algae. A large amount of it was washed ashore during summertime, decomposed and caused a terrible odor in the coastal region. The cost to remove it created a large financial burden to local communities. We wish to grow alga and bury them in the ocean floor. The feasibility of it requires extensive research in terms of chemical balance, ecological balance and physical implementation.

Before the forum, the method I prepared to propose was CCS of 100 million tons. This, in my opinion, is humans' last resort to reach carbon neutrality. The current annual amount of CO_2_ buried underground is less than 100 million tons, of which 40 million tons are used to improve the efficiency of oil and gas extraction. During the 14th five-year plan, Sinopec reached million-ton capacity of CCS at the Shengli Oilfield that is operating now (Figure 3). The next step of Sinopec is the capacity of 5 million tons of CCS, also aimed at improving extraction efficiency. So, in the short-to-mid term, we should stay on this course. But in the long term, relying on the oil industry to provide CCS as a by-product is not enough. For the purpose of mitigating climate change, the target value of CCS is thousands of times the amount we can get from the oil industry. As we look closely at how to drastically improve the capacity of dedicated efforts of CCS, we see two ruling factors: first is the cost based on technological advancement; second is carbon price. When the carbon price is high, paying to bury is more cost-effective than paying for emission. When the carbon price is low, people would still choose to pay for emission. On the other hand, when CCS technology is advanced, price to bury can be reduced below carbon price then CCS becomes economically viable. The current market price of 1 ton of CCS is between 300 yuan to 600 yuan and I estimate the price may drop to 200 yuan by 2030. Our challenge is to see if we can reduce it even lower than 200 yuan by upscaling capacity and that is why I chose a target capacity of 100 million tons. So that is the price factor. Next issue is location. To that, our answer is exhausted oil fields. For example, the largest oil field in the world, Ghawar Field in Saudi Arabia has an estimated capacity of 10 billion tons and now it is almost exhausted. Potential CCS sites are certainly not limited to the Middle East as there are suitable oil fields in Russia, the US and China. Our Daqing Field has a capacity of 5 billion tons and it may serve as a substantial CCS site when it is exhausted. I also saw similar plans made for oil fields in the Gulf of Mexico. I encourage researchers to pay attention to similar proposals from around the world.

**Figure 3.**
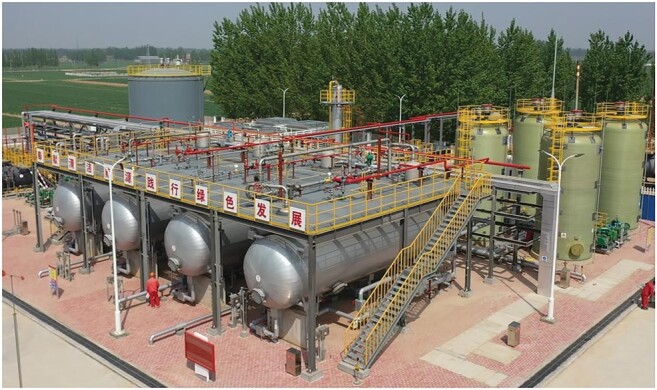
Injection site of million-ton CO_2_ at the Shengli Oilfield (credit: courtesy of Shengli Oilfield).

Another possibility of geoengineering comes from my discussion with meteorologists. When we successfully predict the weather over a weekend or in a week, we may be able to avoid significant damage caused by extreme weather events. But mid-to-long term forecasts, in the range of a few weeks to months, are still very difficult. We all have heard of trials of artificial precipitation to alleviate drought. Is it possible to spread out a focused patch of precipitation over a larger area to reduce its intensity and damage? I think this should also fall into the category of geoengineering. Hopefully, we can see geoengineering has great potential as well as great urgency. Recently, I have noticed a great emphasis on emission reduction toward carbon peaking and carbon neutrality. I think this is seriously misguided. Economic development is a fundamental human right. Only 1 billion people on earth are living in developed societies; 6 billion more are still on the way to live better lives. No one can take that hope away from them. So, from a global point of view, economic development should remain the theme and carbon emission is unavoidable. We should invest in CCS and SRM to reduce CO_2_ and temperature.

**Zhu**: Thank you, Prof. Jin for this exciting introduction of CCS and its important role in humans' response to climate change. Next, we shift directly to the social aspects involved in geoengineering. Prof. Xu, would you like to discuss this topic?

Interdisciplinary research is needed to integrate natural science aspects and social science aspects of geoengineering. We need to involve scholars in such disciplines as philosophy, ethics and international relations in geoengineering research.

— Jianhua Xu

**Xu**: Yes, I am happy to share my research and viewpoints on SRM. A simple analogy may help us understand the complexity of governing the deployment of SRM technologies: SRM is like a prescription drug for an ailing patient. It alleviates symptoms but comes with side-effects. In this regard, it presents a sharp contrast to emission reduction. Any country reduces carbon emissions is beneficial to the whole world. But SRM is quite different in that it provides a collective benefit to the globe but it may affect certain regions negatively. So, it will raise ethical issues regarding common fairness. Basically, deploying SRM technologies is like choosing the lesser of two evils.

In our research report which is a collaborative work of scholars from several countries, we used a risk-risk tradeoff framework (Figure 4) to analyze the pros and cons of deploying SRM technologies, which can serve as a basis for facilitating negotiation and building consensus. In this framework, the target risk is the risks caused by climate change, the intervention is measures or policies for reducing the target risk, and the countervailing risks are the side-effects of the intervention. The basic structure of the framework is not complicated, but implementing the framework is not that easy. Taking stratospheric aerosol injection as an example, one thing to be done in this framework is to determine the amount and frequency of aerosol injection that is equivalent to the dosage of a prescription drug. Different injection amounts are associated with different costs, benefits and side-effects and they will be distributed differently among countries and regions. These all need to be estimated. The illustrative example in our research report treated SRM as a measure in addition to emission reduction, CDR, and adaptation. Our preliminary analysis demonstrated that SRM is expected to quickly reduce the risks associated with temperature rise in most regions of the world, such as reduction in the frequency and intensity of extreme weather events, slowed rise of sea levels, slowed melting of Arctic Sea ice and mountain glaciers. SRM is also predicted to bring unintended countervailing risks as well, such as regional changes of precipitation, increased acid deposition in high latitude regions, stratospheric ozone depletion, and potential shock of sudden termination of SRM.

Centering on SRM, the scientific community has accumulated extensive new knowledge and we keep moving forward. What I want to stress is that deploying such SRM technologies as stratospheric aerosol injection faces situations different from most of the global environmental issues. Existing international treaties mostly prescribe actions that are good for the global society once taken by individual countries or regions, while SRM technologies bring non-negligible side-effects while bringing benefits to the global society. We need to develop global mechanisms for governing research and deployment of SRM.

**Zhu**: Thank you for the description. My follow-up question is that even though we are gaining new knowledge, there still exist substantial uncertainties when we discuss an unprecedented endeavor such as SRM. How should we deal with uncertainties in this area?

**Figure 4.**
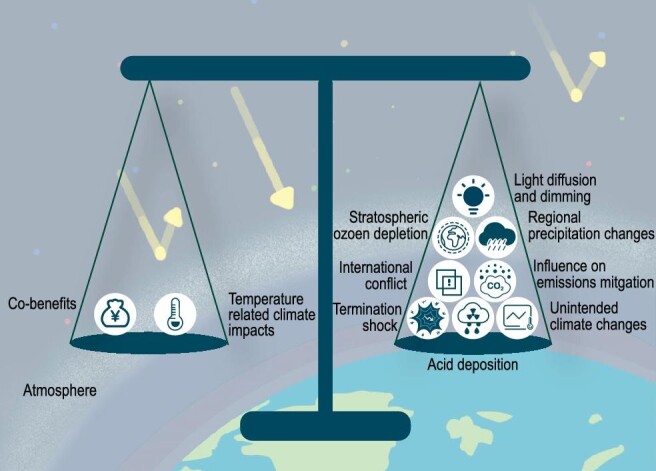
A risk-risk tradeoff framework to analyze the pros and cons of deploying SRM technologies (credit: courtesy of Xu research group).

**Xu**: This is a thorny issue. Uncertainties in science have always been used to support or oppose actions. The proponents of certain technologies may use the uncertainties to their advantage, who may argue that we always need to make decisions amid uncertainties as delayed decisions have consequences. The opponents of SRM also cite the uncertainties to their advantage by arguing that we cannot move forward until we have a better understanding of the uncertainties associated with the technologies. Decision mechanisms need to be developed to build consensus regarding the deployment of SRM. Nevertheless, we need further research on geoengineering. In addition, codes of conduct to guide research efforts are to be developed.

**Zhu**: The last point I want to make is that even though geoengineering is gaining attention overseas, relevant discussion and research in China are still lacking. So, I want to ask all the experts to briefly make a few suggestions to address that. Prof. Liu, would you like to start?

**Liu**: First, domestic researchers should organize proper reviews of progress overseas. This will be highly beneficial to start our own efforts. Second, I think we should take advantage of our efficient governing model and make notable contributions. This will strengthen our position in the global community in addressing issues such as climate change.

**Chai**: Of the three main geoengineering efforts we talked about today: SRM, iron fertilization and artificial intervention of weather, I want to emphasize that we need to clearly distinguish the scientific research efforts from engineering implementations. This has to be made very clear for the public and acknowledge the scientific uncertainties and risks involved.

**Xu**: Interdisciplinary research is needed to integrate natural science aspects and social science aspects of geoengineering. We need to involve scholars in such disciplines as philosophy, ethics and international relations in geoengineering research. The research report that I mentioned earlier was an example of collaborative research of scholars from different disciplinary backgrounds, where natural and social sciences are integrated to discuss the risk-risk tradeoff as well as governance issues regarding the deployment of SRM technologies. In addition, I think the funding agencies in China should support more projects in geoengineering. That would help to boost domestic efforts in this area.

**Jin**: My final suggestion is that the forum discussion we have today should be summarized, distributed and most importantly, expanded. In the future, we may organize larger scale symposia on this and include extensive talks and open discussions. Hopefully, we will be able to gradually increase the awareness of this topic and the influence of this research community and make substantial progress.

As scientists, we have an obligation to investigate possible geoengineering efforts that are feasible and effective to implement and only present manageable side effects.

— Tong Zhu

**Zhu**: I’d like to thank all panelists today participating from around China and abroad. As we all stressed, geoengineering is an important and far-reaching topic. In the course of climate change, we are now witnessing the impact of rising temperature of about 1.5°C around us. And we now know even if we reach the goals of carbon peaking and carbon neutrality, the projected temperature rise may be more than 4°C. Now we face the question of if it is possible to mitigate climate change relying solely on emission reduction. As scientists, we have an obligation to investigate possible geoengineering efforts that are feasible and effective to implement and only present manageable side-effects. Now we discovered possibilities such as limited scale CDR, SRM and iron fertilization. In order to move beyond the stage of hypothecation, further discussions and dialogues are imperative to form a consensus. As Profs Jin and Liu concluded, more forum discussions such as this should be held in a larger scale. We all look forward to working with NSR in the future to bring this exciting topic to a broad audience. Once again, I thank all of you for your contribution today.

## Notes

(朱禾) is a science and news editor at the editorial office of NSR in Beijing, China.

